# Charity's Much-Injured Name

**Published:** 1890-12-20

**Authors:** 


### The Doctor's Armchair.

### "CHARITY'S MUCH-INJURED NAME."

"Come all ye, too, who defame
Charity's much-injured name!
By the lies you circulate,
By your sanctimonious prate,
By the trust which you abuse,
By the funds which you misuse,
By the children you maltreat,
By the victims whom you cheat,
By your smug and unctuous air,
By the fiendish frauds you dare,
Now appear! appear!"

Mr. Labouchere assumes the rôle of the arch-hypnotiser in this year's Christmas number of *Truth.* By means of incantations and hypnotic powers he summons all the maleficent spirits of the age from the vasty deep, and among those evil powers he calls up certain of the representatives of philanthropy, and bestows upon them a paternal adjuration in the briskly flowing lines we have quoted at the beginning of our present talk.

It is not at all bad for Labby, and we can quite imagine how he enjoyed penning, or at any rate editing, these and other sentiments of a similar kind. In so far as there is truth—not Mr. Labouchere's *Truth,* but truthfulness—in the words, all honest folks will be grateful for them. There may be in London men of bankrupt character and unfeeling heart who use the name of Charity to conjure with, and who manage to divert the lion's share of the proceeds to their own use. If Mr. Labouchere could apply to these some punishment more practically effective than mere words of denunciation he would deserve still more approval at the hands of honest men. "By the lies you circulate; by your sanctimonious prate." True, Sir, we cannot deny altogether that some who use the name of charity circulate lies. They do it from choice, and deliberately: it is a matter of business with them: they find it to pay. They do not consider themselves infamous; the more's the pity. But they are infamous; and their infamy is quite unnecessary. Honesty would do at least as well, probably better. Those who condescend to circulate lies will not hesitate to season them with sanctimoniousness. What shallow and stupid fools they are who think that where a lie cannot succeed by itself, it is sure to accomplish its end when hypocrisy rides on its back!

"Trust abused: funds misused!" Charges like these are not quite the Christmas gifts which the representatives of doubtful charitable institutions receive with pleasure. But the question is—are they the kind of Christmas greetings that certain of those institutions have merited? It may be that they are; and if so it is well that truth has been spoken, and spoken without reservation, and to the right people. But we would like to see the particular institutions, if there be such, "named" and specified. Denunciations at large are worth very little. "Trust abused!" That is where the baseness and the crime lie. Funds may be misused in ignorance and inexperience, and without evil intent. But a certain number of those who handle charitable moneys, it seems, are cheats and knaves. Instead of being where they are in their snug and comfortable berths, they would be oakum-picking if they had their due. It is a pity that in this case again Mr. Labouchere cannot name the offenders, and use something stronger than words.

Thus far we are disposed to go with Mr. Labouchere. Though we do not regard him altogether as an inspired prophet, or as a man whose peculiar sanctity is such as to entitle him to speak to his fellow-sinners from the lofty pedestal of an absolutely spotless perfection, yet in that he may have uttered truths that needed to be proclaimed, we, and the representatives of *bona fide* Medical Charities, may add our grateful Amen. But what does he mean when he says: "By the children you maltreat?" This looks like a line put in for purposes of rhyme; and as rhyme it may be unobjectionable; but we do not believe there is any reason in it. Mr. Labouchere assumes the right to denounce other people when they are wrong: does he know of any just cause or impediment why he himself should not stand in the pillory if he has ventured into paths that are not honest and fair? Now either "children" are "maltreated" at hospitals and other charitable institutions, or they are not. We deny that they are; and we challenge Mr. Labouchere to furnish his proofs to the contrary. If he does not know of any actual cases of the maltreatment of children by "charitable" folks, what is his motive for penning a base charge? And if he does know of any such cases, where is his superior loyalty to virtue and paternal kindness in withholding his knowledge from the public?. There are other things that are profitable as well as the offices of so-called charitable institutions. There may be a considerable income attached to the proprietorship of *Truth;* and the profits of Christmas numbers may sometimes be increased by paragraphs well flavoured with piquant seasoning; and if plain truth be, occasionally, like fennell, a little tasteless, there may be pecuniary advantages in using a dash of the literary cayenne of exaggeration.

There is no reason whatever why Mr. Labouchere should have all the cynicism and satire entirely to himself. What is sauce for the goose we know to be sauce for the gander, and probably it might not be very unsuitable even for a milk-white swan. Is Mr. Labouchere a milk-white swan? Even if he be, and his Christmas number were his death song, we should still decline to believe in the maltreatment of children at charitable institutions, except on the production of evidence of the amplest and most convincing kind.

Mr. Labouchere does not believe much in measure. A half truth may be a whole lie. The charge against charities which we have quoted at the beginning of our article is a half truth which will have the effect of a whole lie upon many people's minds. It affirms that some who speak in the name of charity are base persons, and that is true. But what it does not affirm is the proportion of the base to the excellent. The "cheats" and the "shams" denounced by Mr. Labouchere are but as the one or two black sheep in a white flock of hundreds. Are the institutions represented by the hundreds to be branded and defamed merely that the one or two individuals may not fail of punishment? Are all other classes of men, weekly editors and members of Parliament included, so uniformly perfect that not even one black sheep is to be looked for in the army of charity? It is a bad world, we admit, when we put our spectacles of cynicism on; but it is a good world, we perceive, when we look round with eyes of tolerant brotherhood. The present is a season for the use of such eyes. Even those who are attacked may be permitted for a few days to escape from the scrutiny of the microscope. Such persons are found to be exceedingly like other men when scientifically studied in detail; and they ought not, in fairness, to be quite excluded from the kindly judgments to which Christmas disposes the wholesome and unpharisaical mind. Moreover, charitable institutions, and particularly hospitals, and their patients, are accustomed to bask in the warmth of a genial prosperity at Christmas time. Even Mr. Labouchere himself would hate to play the part of the dismal fog, and to envelop the unfortunate men and women whom sickness has laid its hand upon in its cold and depressing gloom, by depriving them of their keenly anticipated share of the good things of the season. In spite of his words of denunciation, we know what his deeds are to the ten thousand children to whom he is both Santa Claus and Father Christmas in one. He will forget, and all other generous people will forget, that there have been one or two "Charity" scandals during the year; but they will not forget that of all our country's noble charities the hospitals are the noblest; and that at Christmas time the hospitals have never yet asked in vain for the generous bounty of the rich and the kind of heart.

